# A quantitative process evaluation of a feasibility randomised controlled trial of a co‐designed cognitive behavioural therapy intervention for people with type 1 diabetes and disordered eating (steady trial): Auditing treatment integrity and delivery

**DOI:** 10.1111/dme.70124

**Published:** 2025-09-12

**Authors:** Amy Harrison, Natalie Zaremba, Jennie Brown, Divina Pillay, Jacqueline Allan, Rachael Tan, Janet Treasure, Khalida Ismail, Marietta Stadler

**Affiliations:** ^1^ Department of Psychology and Human Development University College London, Institute of Psychiatry London UK; ^2^ Department of Diabetes School of Cardiovascular and Metabolic Medicine and Sciences, Faculty of Life Sciences and Medicine King's College London London UK; ^3^ Department of Psychological Medicine, Diabetes, Psychology and Psychiatry Research Group King's College London London UK; ^4^ Department of Medical Psychology Radboud University Medical Center Nijmegen The Netherlands; ^5^ Department of Diabetes King's College Hospital London UK; ^6^ Institute of Psychiatry, Psychology and Neuroscience King's College London London UK

**Keywords:** cognitive behavioural therapy, disordered eating, eating disorders, process evaluation, randomised controlled trial, Type 1 diabetes

## Abstract

**Aims:**

Safe management of people with Type 1 diabetes and Eating Disorders study (STEADY), a complex psychological intervention, defined by the Medical Research Council as involving multiple interacting components and individualised delivery, is a treatment designed for people with Type 1 diabetes and mild‐to‐moderate disordered eating (T1DE) which integrates cognitive behavioural therapy (CBT) with diabetes education. STEADY was previously tested in a feasibility randomised controlled trial (RCT), and the purpose of this work was to maximise trial learning to support future scaling up of STEADY in a multi‐site RCT.

**Methods:**

This study addressed three research questions: (1) Which STEADY toolkit tools were used in the intervention, and at which point? (2) To what extent was treatment delivered as intended, reflecting the minimum competency (≥3) on the Cognitive Therapy Rating Scale (Revised; CTS‐R)? (3) How long did it take to deliver the STEADY intervention?

**Results:**

A range of STEADY tools were used during the trial; the five most frequent tools were CBT formulation (72 uses), behavioural experiments (47 uses), thought records (43 uses), goal setting (40 uses) and understanding emotions and ‘riding the wave’ (40 uses). The CTS‐R mean score was 3.81 ± 0.74, indicating competent adherence to CBT. Mean time to completion was 153.3 days (SD = 73).

**Conclusions:**

When scaling up for a multi‐site RCT, some participants may need greater flexibility regarding timing to access all STEADY sessions. STEADY can be personalised through its toolkit‐based approach, and therapists should be mindful and trained in the range of tools available.

**What's new?**

- STEADY integrates CBT with diabetes education to treat disordered eating occurring in the context of Type 1 diabetes.
- This research found the CBT intervention delivered in STEADY met the minimum level of competence on the CTS‐R, suggesting high fidelity of the intervention.
- The most used therapy tools were CBT formulation, behavioural experiments, thought records, goal setting and understanding emotions/emotion regulation strategies.
- Scaling up STEADY in a multi‐site RCT should consider the training level of, and supervision provided to clinicians to ensure high fidelity, and give participants and therapists sufficient time to complete the intervention.

## 1 INTRODUCTION

We developed the Safe management of people with Type 1 diabetes and EAting Disorders studY (STEADY) intervention, using experience‐based co‐design^1^ between 2018 and 2021, and delivered^2^ and evaluated its feasibility and safety^3^ between 2022 and 2024. This individual, 12‐session treatment integrates cognitive behavioural therapy (CBT) with diabetes education for adults with Type 1 diabetes and mild‐to‐moderate disordered eating (T1DE). STEADY is underpinned by a cognitive behavioural model of T1DE^4^, ^5^ and provides the therapist (a diabetes educator trained in CBT or a clinical psychologist with a basic understanding of Type 1 diabetes) with a toolkit, meaning treatment can be personalised to the individual's therapy goals and is delivered embedded in a multidisciplinary (diabetes and mental health specialists) setting. STEADY therapy sessions also include a health check‐in as part of the agenda, which may involve prompting participants to check their blood glucose, blood ketones, or share their continuous glucose monitoring device download to inform the session, which is a unique integration of the physical health aspects of diabetes into the CBT session. The STEADY intervention was tested in a feasibility randomised controlled trial (RCT) including 40 people with T1DE (38 women, 1 man, 1 trans man) (37 White, 1 White/Asian, 1 Black; 39 ± 11 years old, diabetes duration 22 ± 15 years, HbA1c 9.1 ± 2.6%), compared with treatment as usual.^3^

The Medical Research Council^6^ states that process evaluations are an essential part of designing and testing complex interventions and involve looking inside the “black box” to see what happened in the study and how this could affect outcomes. This goes beyond addressing whether or not the intervention was effective, and advances knowledge by answering relevant questions like *why* the intervention did or did not work, and how it might be better optimised in future.^7^ This can involve exploring the receipt, setting, implementation and meaning of the results using quantitative and qualitative methods.^8^ Having completed the STEADY feasibility RCT, we wanted to develop our learning further through a quantitative process evaluation involving an audit of session delivery and the STEADY toolkit, and measurement of treatment fidelity. A qualitative process evaluation exploring participant experiences of receiving the intervention and healthcare professionals' experience of delivering the intervention is in progress and will be discussed in a future publication.

The first research question was which STEADY tools were used in the intervention, and at which point in each person's treatment? To address this, we conducted an audit of the frequency of STEADY tools, and investigated when the tools were used during the 12 treatment sessions offered.

The second research question was to what extent the treatment was delivered as intended? To address this, we used the Cognitive Therapy Rating Scale (Revised) (CTS‐R)^9^ to understand whether the delivered treatment was consistent with its constituent intended interventions and in keeping with its tenets, known as treatment fidelity.^10^ Therapist adherence to a treatment model is a necessary condition for competent delivery of an intervention^11^, ^12^ and ideally exists alongside skilful adaptations of the intervention for the individual trial patient.^13^ Treatment fidelity here was operationalised as rated sessions meeting the minimum competency on the CTS‐R (≥3).

The third research question was, how many days did it take to deliver the STEADY intervention? As the study protocol^2^ planned for 12 individual treatment sessions to be completed in 6 months, to address this research question, we audited the number of days taken to conclude the delivery of the STEADY treatment, to learn how much time might be needed to complete treatment if STEADY were scaled up and delivered across a multi‐site RCT.

## 2 METHODS

### 2.1 Design

These process evaluation analyses use an observational design. Ethical approval was obtained from the East of England–Essex Research Ethics Committee (21/EE/0235).

### 2.2 Participants

This quantitative process evaluation involved the 20 participants randomised to the experimental arm of the STEADY feasibility RCT. Demographic data are provided in Table 1.

**TABLE 1:** Demographic characteristics of trial participants included in the process evaluation.

	STEADY (*n* = 20)
Sex at birth	
Female	19 (95%)
Male	1 (5%)
Gender	
Female	18 (90%)
Male	2 (10%)
Age (years)	35.5 (30.5–48.8)
Ethnic origin	
White – English/Welsh/Scottish/Northern Irish/British	17 (85%)
White – Gypsy or Irish Traveller	1 (5%)
White‐ any other background	0 (0%)
Black – Caribbean	0 (0%)
White – English/Welsh/Scottish/Northern Irish/British	17 (85%)
Diabetes duration (years)	16.0 (8.2–30.8)

*Note*: Data are *n* (%). Please refer to Stadler et al.^3^ for clinical data on the sample.

### 2.3 Measures

#### 2.3.1 STEADY tool audit

Eighty distinct items form the STEADY toolkit manual (Table of Contents provided in Supplementary Material), and these are referred to as ‘STEADY tools.’ The toolkit involves patient‐ and clinician‐facing items, including activities, handouts, and worksheets. Here, we will describe which tools were used, at what frequency, and when they were deployed within the intervention. These data had been entered into the trial database on RedCap® during the trial. Data were extracted retrospectively (after trial completion) from RedCap® participant records. This was completed by using the Data Exports function. A new custom report was created to define the appropriate fields for the dataset (tools used, which had been entered in a free‐text box, and session number, which was a field set up in the database for each participant, with a minimum of 0 and a maximum of 12). Filter logic was used to include only participants randomised to receive STEADY, and Record Ordering was selected so that the data were organised by participant and session number.

#### 2.3.2 Treatment fidelity

The Cognitive Therapy Rating Scale Revised (CTS‐R)^9^ measures treatment integrity and therapist competence in delivering cognitive therapy techniques and is widely used in CBT interventions across trials in outpatient and community settings.^14^ This valid and reliable scale^15^ consists of 12 items rated on a 7‐point Likert scale ranging from 0 (absence of feature, or highly inappropriate performance) to 6 (excellent performance, or very good even in the face of patient difficulties). The CTS‐R provides competency categories for the numerical outcomes, with 0 to 1 described as ‘incompetent,’ 1 to 2 as ‘novice,’ 2 to 3 as ‘advanced beginner,’ 3 to 4 as ‘competent,’ 4 to 5 as ‘proficient’ and 5 to 6 as ‘expert.’ A rating of 3 reflects a minimally acceptable level of competency, indicating the therapist is delivering a therapy that adheres to the model and is competent, but some problems and/or inconsistencies remain. Higher scores indicate greater adherence to the CBT model and higher therapist competence in employing CBT techniques. The CTS‐R was used to measure treatment fidelity at the end of the trial. It is also a supervision tool used to support continued professional development and identify and correct therapist drift from the treatment protocol^16^ and was also used in this way in the therapy supervision provided by AH, a clinical psychologist to JB, a Diabetes Specialist Nurse and Cognitive Behavioural Therapist in this trial. JB has extensive experience in Type 1 diabetes and has a Post‐Graduate Diploma in CBT. AH is a Clinical Psychologist with 12 years' post‐qualification experience in specialist inpatient and outpatient eating disorder treatment; she has trained in CBT and is a CBT trainer.

Therapy sessions were recorded using the Zoom audio recording function. Participants consented to this optional process evaluation data collection separately from the main trial consent process, having received an information sheet. The rater listened to the full middle session recording (50 minutes). We aimed for session 6, the mid‐point of the intervention. If this recording was not available, due to participant drop‐out/withdrawal, or recording failure, the preceding, nearest available session was selected. The CTS‐R was completed by a second independent rater (DP) for 27% of available recordings (*n* = 4) to assess inter‐rater reliability. The inter‐class correlation coefficient, a measure of the consistency and absolute agreement of the raters, was 0.81, indicating excellent inter‐rater reliability.

#### 2.3.3 STEADY session audit

Session number and date were extracted from RedCap® participant records.

### 2.4 Data analysis

#### 2.4.1 STEADY tool audit

To graphically represent which tools were used and when during each person's treatment, tools were categorised based on their type: general CBT tools, behavioural interventions, cognitive interventions, psychoeducation, outlined in Table 2.

**TABLE 2:** Categorisation of STEADY (Safe management of people with Type 1 diabetes and EAting Disorders study) Tools.

**General Cognitive Behavioural Therapy Tools**
Introduction to cognitive behavioural therapy and STEADY
Formulation
Goal Setting
Relapse planning and prevention

General	Diabetes‐specific
**Behavioural Interventions**	
Behavioural experiment	Insulin titration
Urge or emotion surfing	
Breathing exercises	
Building self‐compassion	
Looking out for good things	
Managing difficult emotions	
Looking out for good things	
**Cognitive Interventions**	
Thought records	Acceptance of diabetes and weight
Fear of weight gain	Factors influencing blood glucose
Thinking styles	Perfectionism and diabetes
Thought or fact	Thoughts about insulin and diabetes
Values exercise	
Pie charts	
**Psychoeducation Tools**	
Understanding perfectionism	Sick day rules
Understanding urges and emotions	Exercise and diabetes
	Hypoglycaemia treatment plan
	Hypoglycaemia and driving
	Blood glucose scanning and understanding real‐time glucose monitoring data
	Factors influencing blood glucose

*Note*: Behavioural experiments are listed in the general category but could focus on aspects of mental health or diabetes self‐care. Similarly, though thought records are listed in the general category, they could of course involve thoughts about diabetes.

#### 2.4.2 Treatment fidelity

A total score and mean score, derived from the 12 CTS‐R items, were computed for each rated recording. Frequencies/percentages for the competency categories ratings were produced.

#### 2.4.3 STEADY session audit

While the STEADY study protocol^2^ states participants have completed the intervention if they have attended ≥6 sessions, here, all participants were included to provide a broader overview of the time needed to reach the conclusion of the delivery of the experimental treatment arm.

## 3 RESULTS

### 3.1 STEADY tool audit

**FIGURE 1:**
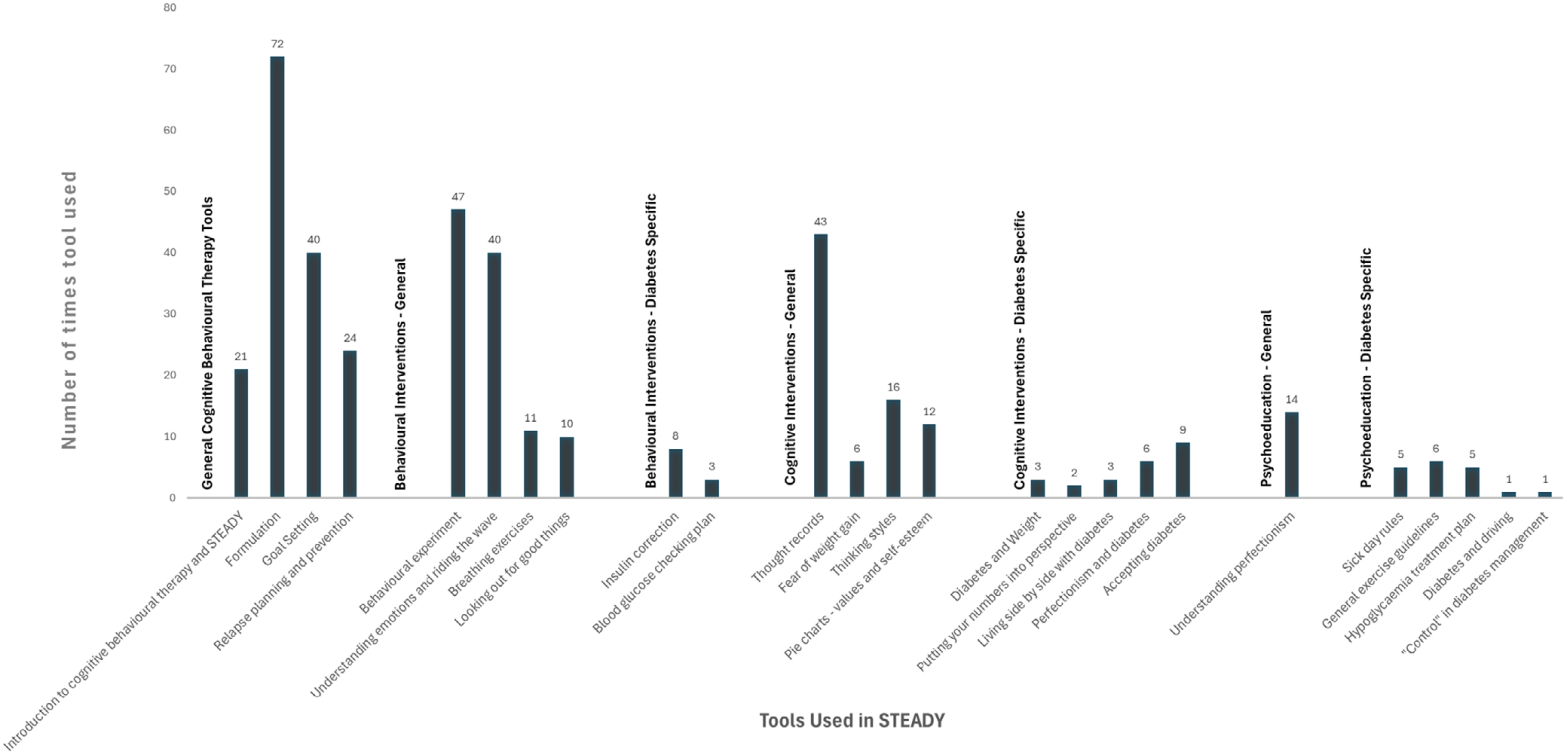
Frequency of STEADY tool use across the STEADY intervention.

Figure 1 provides data on tool‐use frequency across all sessions delivered to the 20 participants randomised to receive STEADY treatment.

Twenty‐five distinct tools were recorded on RedCap as being deployed in sessions. The average frequency of tool use was 13.6 (SD = 16.7). Generally, psychoeducation‐based tools were used less frequently than the cognitive and behavioural intervention tools. The first three tools in the toolkit (hypoglycaemia treatment plan, sick day rules and crisis plan) were already deployed at the baseline clinical assessment to all 40 participants received prior to randomisation and were not captured in this analysis. The five most frequently used tools were formulation (72 uses), e.g. *the 5 aspects task for the STEADY formulation* tool, behavioural experiments (47 uses), e.g. the *behaviour experiments worksheet* tool, thought records (43 uses), e.g. the *thought diaries* tool, goal setting (40 uses), e.g. the *setting therapy goals* (*SMART goals*) tool, and understanding emotions and riding the wave (40 uses), e.g. emotion/urge surfing.

**FIGURE 2:**
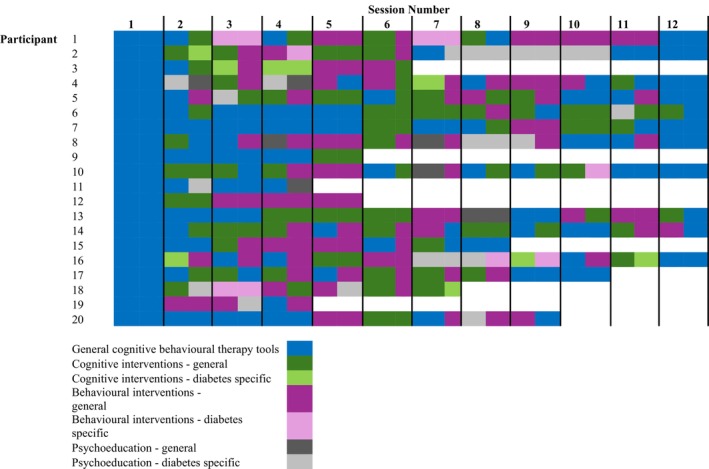
STEADY tools used across the intervention by each participant. White indicates no tools recorded for reasons such as withdrawal, exclusion or study closure. Participant on the Y axis refers to each of the 20 individual participants randomised to receive treatment, and Session Number on the X axis refers to each of the 12 individual treatment sessions made available to participants receiving treatment.

Figure 2 provides data on the timing of tools used within the STEADY intervention for each STEADY participant.

### 3.2 Treatment fidelity

The final sample consisted of 15 participants. Five (25%) participants randomised to receive the STEADY treatment did not consent for their sessions to be recorded or for their data to be used in this component of the process evaluation.

Table 3 provides the outcome data for the CTS‐R.

**TABLE 3:** Therapist competence ratings on the cognitive therapy rating Scale – revised (CTS‐R) for sessions conducted with STEADY (Safe management of people with Type 1 diabetes and EAting Disorders study) Study Participants.

Session with Participant:	Session number															
	1	2	3	4	5	6	7	8	9	10	11	12	Total Score	Mean score	Cognitive Therapy Rating Scale Revised Competency Categorisation	Were the STEADY tool(s) reported evident during the recording?
Participant 1	2	5	3	3	3	2	5	2	2	5	2	3	37	3.08	Competent	Yes
Participant 2	2	5	4	5	4	2	2	3	3	3	2	6	41	3.42	Competent	Yes
Participant 3	5	5	6	5	5	4	6	5	6	5	5	5	62	5.17	Expert	Yes
Participant 4	3	4	3	3	4	4	3	1	2	2	3	3	35	2.92	Advanced beginner	Yes
Participant 5	2	4	4	2	5	2	3	4	4	2	2	2	36	3.00	Competent	Yes
Participant 6	3	3	2	2	5	2	3	2	3	2	2	2	31	2.58	Advanced beginner	Yes
Participant 7	4	5	5	5	5	2	3	3	4	4	3	3	46	3.83	Competent	Yes
Participant 8	2	4	5	5	5	3	4	2	3	5	2	3	43	3.58	Competent	Yes
Participant 9	5	6	5	5	5	2	3	2	2	3	5	5	48	4.00	Proficient	Yes
Participant 10	5	6	6	6	5	3	5	3	5	4	5	5	58	4.83	Proficient	Yes
Participant 11	5	4	4	4	4	4	3	3	5	4	5	5	50	4.17	Proficient	Yes
Participant 12	5	5	5	5	5	3	3	2	5	4	1	2	45	3.75	Competent	Yes
Participant 13	5	5	4	5	5	5	5	5	4	4	4	5	56	4.67	Proficient	Yes
Participant 14	5	4	3	5	3	2	4	4	4	5	4	5	48	4.00	Proficient	Yes
Participant 15	5	3	5	5	5	4	5	3	5	5	2	3	50	4.17	Proficient	Yes

*Note*: 20 participants were randomised to receive STEADY, and 15 gave consent for their sessions to be recorded and the data used for this component of this process evaluation. CTS‐R scores reflect therapist competence in delivering CBT during a recorded session conducted with the participant listed. Ratings were based on a single mid‐point session (usually session 6) per participant, where available.

The mean score across the 12 items was 3.81 (SD = 0.74), indicating overall competent adherence to CBT, with a minimum mean score of 2.58 (advanced beginner) and a maximum mean score of 5.17 (expert level skill). None of the sessions (0%) were at the incompetent or novice level. Two (13.33%) were at the advanced beginner level, 6 (40%) were at the competent level, 3 (40%) were at the proficient level, and 1 (6.67%) was at the expert level.

An additional check was applied to investigate whether the tools reported being used by the clinicians were evident in the session recording revealed full consistency.

### 3.3 STEADY session audit

**FIGURE 3:**
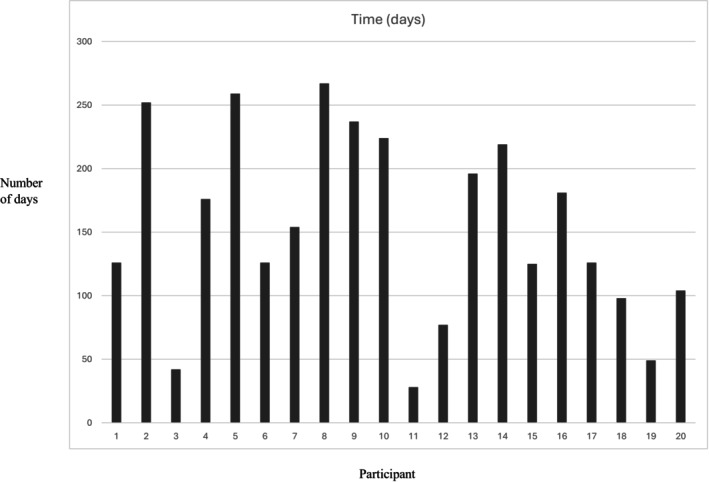
Number of days taken to deliver 12 sessions of STEADY for each STEADY participant.

Figure 3 outlines the number of days taken to complete 12 sessions of STEADY treatment for each participant.

It took a mean of 153.3 days (SD = 73) for all 20 participants to complete treatment (attend ≥6 sessions), or end treatment due to drop‐out/withdrawal from the study/because the trial ended (range: 28 and 267 days). Seven participants (35%) needed >6 months to complete treatment due to ill health, needing to prioritise acute medical needs and significant life events, or because of a strong clinical rationale to alter the time between sessions, for example, to allow sufficient time to complete a behavioural experiment.

## 4 DISCUSSION

This observational, quantitative process evaluation study sought to address three research questions: Which STEADY toolkit tools were used in the intervention, and when; to what extent was treatment delivered as intended; and how long did it take to deliver the STEADY intervention? These questions were addressed through an audit of STEADY tool usage, through measuring treatment fidelity using the CTS‐R and through a session audit.

Formulation, behavioural experiments, thought records, goal setting and understanding emotions and ‘riding the wave’ (surfing urges and riding out emotions) were, respectively, the five most frequently used components of the STEADY intervention. They may be the core components of the intervention. They may also have been the tools that were more familiar to clinicians and were relied on more frequently in sessions. Another perspective is that, guided by participants' formulations, these were the most appropriate tools to help individuals reach their treatment goals. In STEADY, ‘formulation’ refers to the shared cognitive behavioural model co‐constructed with each participant to understand their difficulties. From this formulation, ‘treatment goals’ are collaboratively derived as the overarching aims of therapy. Participants were invited to set their own personal goals meaningful to their own intentions for treatment, which in some cases referred to mental health outcomes, such as changing thoughts and feelings around weight and shape, and in other cases, to diabetes self‐care variables, like evoking a change in Hba1c. ‘Goal setting’ then refers to the process of breaking these goals down into specific, measurable behavioural targets agreed during sessions, often using tools such as SMART goals worksheets. While overlapping in purpose, each serves a distinct function in structuring the personalised therapy process. So, this finding may also reflect good adherence to the cognitive model – all participants had an individual formulation, which, evidenced by the data on tool‐use timing, took place at the start of treatment (and was re‐visited in most cases throughout the intervention). Participants also set goals towards the start of treatment, which were reviewed at later points in treatment, and they received support around relapse planning and prevention towards the end of treatment. The data suggest that the middle section of treatment involved multiple behavioural interventions such as the frequently used understanding emotions and developing emotion regulation strategies, behavioural experiments and insulin titration work, alongside cognitive interventions like thought records. The frequent use of these core CBT interventions, which involved participants actively changing diabetes self‐care and disordered eating behaviours, and developing different cognitions about eating, shape, weight and diabetes, may be the underpinning mechanisms of change that account for the improved biological and psychological outcomes observed in those who received STEADY treatment compared to the control group.^3^

While the STEADY intervention draws on standard CBT tools, these were adapted to address the specific emotional and behavioural patterns seen in T1DE. For example, thought records were commonly used to examine diabetes‐specific cognitions, such as ‘taking insulin will make me gain weight’ or ‘high glucose means I'm a failure’, which often underpinned restrictive eating or insulin omission. Behavioural experiments were designed to test the outcomes of new diabetes self‐care behaviours, for instance, adjusting insulin doses or checking glucose after a challenging meal, to reduce fear of weight gain or dysglycaemia. Emotion regulation strategies like ‘riding the wave of emotion’ were contextualised to help participants cope with diabetes‐related distress, burnout or negative emotions about shape and weight, for example. These adaptations emerged through our co‐design process^1^ and were embedded in the STEADY toolkit to ensure relevance and applicability to everyday diabetes self‐care.

In STEADY, the frequent use of core CBT components such as formulation, behavioural experiments, and thought records reflected not only adherence to the CBT model but also their relevance to the specific challenges of T1DE. Participant formulations often focused on the interaction between disordered eating behaviours and diabetes self‐care, for example, how fear of weight gain might lead to insulin omission, or how guilt following a binge could trigger restrictive eating. These personalised, shared understandings informed bespoke treatment goals, such as reducing shame around blood glucose readings or increasing flexibility in eating while maintaining glycaemic stability. CBT tools were then selected and adapted to support these goals, allowing for targeted interventions that addressed both psychological and diabetes‐specific concerns. By linking cognitive and behavioural patterns with diabetes management behaviours, these tools likely represent key mechanisms of change within the STEADY intervention.

This meant that STEADY is a personalised intervention that, having developed a shared formulation with the individual, involves the clinician selecting appropriate tools to use throughout the intervention, across the categories of behavioural and cognitive interventions, and psychoeducation, in the domains of diabetes self‐care and mental health. On average, each tool was used 13.6 times, and the high standard deviation of 16.7 indicates significant variation between the most and least utilised tools. Tool use was a topic we regularly reflected on in supervision. We were mindful of the range of tools we had available to support participants and reflected that training larger cohorts of clinicians to deliver STEADY would involve needing time to familiarise clinicians with the co‐designed toolkit. However, the toolkit contains only interventions which would be familiar to clinicians trained in CBT or diabetes education, but the tools are optimised through our co‐design work^1^ to tailor them to the complex interactions that develop between diabetes self‐care and mental health factors.

Generally, psychoeducation‐based tools were used less frequently than the cognitive and behavioural intervention tools. This may reflect the existing expertise and skill base of the volunteer sample who participated in the STEADY feasibility RCT, which meant these tools were less needed, perhaps. However, this may be best explained by the initial diabetes education session delivered before the psychological intervention, which was part of the study protocol and meant less therapy time was needed for upskilling participants in diabetes self‐care.^2^

The mean score across the 12 CTS‐R items was 3.81 (SD = 0.74). It is above the minimum level of competence indicated by the scale and means participants received an intervention, which adhered to the treatment approach (CBT). The score is similar to that reported in a large RCT using CBT to treat depression in adults (4.15)^15^ and mirrors the competence observed in CBT interventions for anxiety.^17^ This was achieved by clinicians with post‐graduate training in CBT (e.g. JB is a Diabetes Specialist Nurse with a Post‐Graduate Diploma in CBT, and AH is a Clinical Psychologist) who were the therapists delivering STEADY. We have developed specific diabetes CBT training modules to support the diabetes and psychology workforces to have the knowledge and skills needed to deliver integrated diabetes and mental health interventions like STEADY (see https://www.kcl.ac.uk/short‐courses/cognitive‐behavioural‐therapy‐for‐diabetes‐module‐1). It is important to note that alongside this specialist training/expertise, the intervention was delivered within a multidisciplinary team setting with weekly clinical supervision with the study Diabetologist, Consultant Psychiatrist and Trial Manager to discuss additional issues, safety risks and diabetes care, and also involved weekly therapy supervision. These factors are important components of safety and effectiveness in scaling up to a multi‐site RCT.

The mean time to complete (or drop out, be excluded, or withdraw from) the 12 STEADY sessions of 153 days is within the planned 6‐month period designed for treatment completion stated in the trial protocol. For most participants, 6 months provided sufficient time to complete treatment. However, some (*n* = 7, 35%) needed additional flexibility and required over 6 months to complete treatment, with 267 days (almost 9 months) being the maximum. Reflecting on our clinical experience of delivering the STEADY intervention, additional time was needed due to necessary interruptions caused by life events, which meant treatment needed to be paused temporarily; because ill physical health meant participants requested sessions were postponed/rescheduled; because we made clinical decisions to alter the gap between sessions – the rationale often being to give the person sufficient time to complete a behavioural experiment; or to support participant safety – for example, to enable liaison with the diabetes team, all of which increased the time between the planned weekly meetings. On reflection, session scheduling was more flexible than clinical trials typically expect,^18^ which felt appropriate given the complex comorbidity we were seeing to treat. In supervision, we also reflected on how much change was possible in 12 sessions and whether this was sufficient to produce meaningful change from the perspective of the participant, for example, where insulin titration was involved. This is currently being investigated further from the perspectives of participants and healthcare professionals in the qualitative process evaluation in progress.

A number of limitations, clinical implications and future questions emerge from this process evaluation. The data on tool usage are somewhat limited by how tools were recorded in RedCap. In hindsight, we should have formalised our way of recording STEADY tool usage using a drop‐down box option listing the STEADY tools rather than free text, and we will improve this in a larger RCT with a drop‐down menu on RedCap®. For example, intervening using a particular tool could involve multiple items from the STEADY toolkit, and we did not record this in a granular way.

One limitation is the missing CTS‐R data for five participants (25%) in the intervention arm, who did not consent to session recording. While we found no clear demographic or clinical differences between participants with and without CTS‐R data, it remains possible that therapist competence or session dynamics differed in unmeasured ways. This may introduce bias in our estimates of treatment fidelity, and we will aim to mitigate this risk in future studies by offering enhanced options for secure and confidential recording and by examining predictors of consent to recording.

Some participants used multiple initial sessions for formulation. Supervision was used to explore more rapid movement to behaviour change and cognitive interventions; however, some participants needed more time, given the complexity of the comorbidity. We did not measure concepts like therapeutic alliance, but reflected in supervision that allowing some flexibility in the time allocated to this task felt important for our working relationship. We were working with people who (mostly) had not received integrated diabetes and psychological care and, like the lived experiences informing our CBT model of T1DE,^4^, ^5^ had difficult experiences of healthcare services and significant comorbidity (see^3^ in Table 1) In CBT for eating disorders (without diabetes), research suggests that although a strong early therapeutic alliance is beneficial, early symptom improvement is a stronger predictor of positive treatment outcomes later in therapy.^19^ A larger multi‐site RCT will provide opportunities to measure the importance and impact of the alliance in STEADY. The modular approach to treatment emerged out of our initial co‐design work^1^ and is in keeping with personalised or precision medicine approaches, including the personalised care pathways developed in eating disorder treatment,^20^ which we think is particularly important given the significant comorbidity we have identified in this patient group.^3^ With a larger sample, we will be able to explore further the mechanisms of change and examine the impact of the different types of tools used in treatment, which will assist with further optimisation of the therapy. Specifically, in a larger multi‐site RCT, we will be able to investigate whether therapist competence (as indexed by CTS‐R ratings) or the relative emphasis on cognitive versus behavioural tools predicts improvements in glycaemic control or reductions in disordered eating symptomatology. These analyses will provide further insight into the mechanisms of change and help to refine the intervention.

In conclusion, STEADY can be delivered, in most cases, within a 6‐month period, and after developing a shared formulation and treatment goals, offers an intervention that can be personalised, helps people to understand and manage difficult emotions, and provides time to plan and prepare for relapse. In the context of T1DM and disordered eating, this included relapse into behaviours such as insulin omission, restrictive eating, binge eating, or avoidance of glucose monitoring. Relapse planning typically involves identifying early warning signs, recognising past triggers (e.g. distress over weight or glucose readings), and reinforcing adaptive strategies developed through the STEADY toolkit to maintain both psychological well‐being and diabetes self‐care. Supervision pairing of a clinical psychologist and diabetes specialist nurse with CBT training worked well to bring together the expertise needed to support people with this complex comorbidity and offered opportunities for learning. We are now seeking funding to scale up STEADY to be delivered and evaluated further within a multi‐site RCT.

## CONFLICT OF INTEREST STATEMENT

None to disclose.

## Supporting information


Appendix S1.
