# Supplementary material for: A quantitative process evaluation of a feasibility randomised controlled trial of a co‐designed cognitive behavioural therapy intervention for people with type 1 diabetes and disordered eating (steady trial): Auditing treatment integrity and delivery
Source: Diabet Med. 2025 Sep 12;42(11):e70124. doi: 10.1111/dme.70124 (PMC12535373; doi:10.1111/dme.70124)
Supplement: Supplementary file 1 — Appendix S1. [file DME-42-e70124-s001.docx]

**Appendix supplemental Tables**

**Supplemental Table 1a. Contents of the STEADY manual.**

| **STEADY tool kit table of contents** |
| --- |
| **1. STEADY participant handheld notes** |
| 1.1 Hyperglycaemia treatment plan/ sick day rules- MDI |
| 1.2 Hypoglycaemia treatment |
| 1.3 Mental health and medical health emergency plan |
| **2. STEADY sessions** |
| 2.1 First session, Introduction to CBT and STEADY |
| 2.2 Setting priorities for treatment |
| 2.3 Understanding my Challenges |
| 2.4 The 5 aspects task for the STEADY formulation |
| 2.5 The maintenance cycle of type 1 diabetes and disordered eating for STEADY formulation- T1DE lived experience and maintenance cycles |
| 2.6 A visual exploration of my values |
| **3. Therapy sessions 2-11** |
| 3.1 Suggested session structure |
| **4. Final STEADY therapy session (Session 12)** |
| 4.1 Plan for further care |
| 4.2 Relapse prevention and understanding my relapse |
| **5. Toolkit for the STEADY therapy sessions** |
| 5.1 Hypoglycaemia vicious cycles |
| 5.2 Insulin omission vicious cycles |
| 5.3 Setting therapy goals (SMART goals) |
| 5.4 Setting treatment goals |
| 5.5 Accepting diabetes |
| 5.6 Living side by side with diabetes |
| 5.7. Diabetes and weight |
| 5.8 A visualisation of your values (Pie chart exercises) |
| 5.9 The meaning of “control” in diabetes |
| 5.10 “Control” in diabetes management |
| 5.11 Putting your numbers into perspective |
| 5.12 HbA1c and diabetes management |
| 5.13 The meaning of “control” in diabetes and disordered eating |
| 5.14 Fear of weight gain |
| 5.14 Feeling distressed about weight – what can I do? |
| 5.15 Blood Glucose checking plan |
| 5.15 Goal setting for blood glucose checking plan |
| 5.15 STEADY helpful and unhelpful diabetes self-care |
| 5.16.Ways to explore your diabetes self-care |
| 5.17 Exploring hyperglycaemia in the STEADY therapy session |
| 5.18 Exploring your experience of hyperglycaemia |
| 5.19 Reflecting on a situation when you had hyperglycaemia |
| 5.20 High blood glucose and complications |
| 5.21 Challenging hyperglycaemia |
| 5.22 Challenging unhelpful self care behaviours |
| **6. Mental health and diabetes** |
| 6.1 Depression and type 1 diabetes |
| 6.2 Anxiety and type 1 diabetes |
| 6.3 Perfectionism and type 1 diabetes |
| 6.4 Thinking styles and type 1 diabetes |
| **7. Developing an individualized treatment plan** |
| 7.1 Developing an individualized Insulin correction plan |
| 7.2 SMART goals for insulin correction plan |
| 7.3 Insulin correction plan worksheet |
| 7.4 Developing an individualized re-introduction of background insulin plan (multiple daily injections) |
| 7.5 Goal setting for the re-introduction of background insulin plan (multiple daily injections) |
| 7.6 Treatment plan for re-introduction of background insulin (multiple daily injections) - Worksheet |
| 7.7 Developing an individualized re-introduction of basal insulin plan (pump) |
| 7.8 Goal setting for treatment plan for basal insulin delivery (pump) |
| 7.9 Treatment plan for basal insulin delivery (pump) worksheet |
| 7.10 Developing an individualized plan for reducing background insulin and increasing rapid acting insulin |
| 7.11 Goal setting for reducing background insulin and increasing rapid acting insulin |
| 7.12 Treatment plan for reducing basal insulin stepwise and increasing rapid acting insulin Worksheet |
| 7.13 Developing an individualized plain for reducing the fear of hypoglycaemia |
| 7.14 Goal setting for the hypoglycaemia stepwise treatment plan |
| 7.15 Fear of hypoglycaemia stepwise treatment plan worksheet |
| 7.16 Purging after rapid acting insulin treatment plan |
| 7.17 Goal setting for purging after rapid acting insulin treatment plan |
| 7.18 Purging after rapid acting insulin treatment plan worksheet |
| 7.19 Binge eating and hypo treatment plan |
| 7.20 Goal setting for the binge eating and hypoglycaemia treatment plan |
| 7.21 Binge eating and hypo treatment plan worksheet |
| 7.22 Understanding emotions and riding the wave |
| 7.23 My STEADY exercise plan |
| 7.24 Smart goal setting for Exercise Plans |
| 7.25 My STEADY exercise plan worksheet |
| **8. Educational material (also available in App)** |
| 8.1 STEADY therapeutic approach |
| 8.2 How to use the STEADY App |
| 8.3 What is type 1 diabetes? |
| 8.4 What is hyperglycaemia? |
| 8.5 Multiple daily injections without structured education- hyperglycaemia (sick day rules) |
| 8.6 Multiple daily injections with structured education- hyperglycaemia (sick day rules) |
| 8.5 Insulin pump therapy without structured education- hyperglycaemia (sick day rules) |
| 8.6 What is hypoglycaemia? |
| 8.7 What is severe hypoglycaemia? |
| 8.8 Hypoglycaemia treatment plan |
| 8.9 Insulin injection technique |
| 8.10 General exercise guidelines |
| 8.11 Breathing exercises |
| 8.12 Diabetes and driving |
| 8.13 Setting SMART goals |
| **9. Worksheets for therapy session and work in between sessions (also available in App)** |
| 9.1 Thought records |
| 9.2 Behaviour experiments |
| 9.3 Behaviour experiments worksheet |
| 9.4 Activity schedule |
| 9.5 Activity schedule worksheet |
| 9.6 Looking out for good things worksheet |
| 9.7 Understanding my difficult emotions and urges, worksheet |
| **10. Recovery and relapse** |
| 10.1 Relapse prevention plan |
| 10.2 Reflecting on a relapse |
